# Role of Glucagon-Like Peptide-1 Receptor Agonists on the Weight Loss of Individuals With Type 2 Diabetes: A Systematic Review

**DOI:** 10.7759/cureus.40448

**Published:** 2023-06-15

**Authors:** Huma Irfan, Namratha Pallipamu, Hadi Farhat, Sai Dheeraj Gutlapalli, Suvedha S Thiagaraj, Twisha S Shukla, Sathish Venugopal

**Affiliations:** 1 Research, California Institute of Behavioral Neurosciences & Psychology, Fairfield, USA; 2 Internal Medicine, Franciscan Health, Lafayette Central, Lafayette, USA; 3 Internal Medicine, California Institute of Behavioral Neurosciences & Psychology, Fairfield, USA; 4 Internal Medicine, University of Balamand, Beirut, LBN; 5 Pediatrics, California Institute of Behavioral Neurosciences & Psychology, Fairfield, USA; 6 Neurology, California Institute of Behavioral Neurosciences & Psychology, Fairfield, USA

**Keywords:** obesity, glucagon-like peptide 1, glucagon-like peptide-1 receptor agonist, weight loss, type 1 diabetes mellitus (t1d)

## Abstract

Obesity is highly associated with type 2 diabetes mellitus (T2DM), both of which can be simultaneously treated with glucagon-like peptide-1 receptor agonists (GLP-1RAs). There are many antidiabetic drugs that can be used for the treatment of T2DM. These drugs have vast modes of action and therapeutic uses. However, they also have different side effects. Some of these side effects, such as weight changes, are sometimes desirable while others are not. This review examines the literature on how GLP-1RA affects both blood glucose and body weight in patients with T2DM and obesity. In this context, GLP-1RA plays a critical part by controlling not only the blood glucose level but also weight. We followed Preferred Reporting Items for Systematic Reviews and Meta-analysis (PRISMA) guidelines and searched for articles from PubMed and Google Scholar databases that reported on T2DM, obesity, and GLP-1RA functions. We selected 13 articles that showed the benefits of GLP-1RA in managing both T2DM and obesity. Our review suggests that GLP-1RA is an innovative therapy that can address both conditions simultaneously.

## Introduction and background

Obesity is a growing public health concern worldwide and is associated with an increased risk of diabetes [1] and its complications leading to morbidity and mortality [1,2]. The prevalence of type 2 diabetes mellitus (T2DM) is high in overweight or obese individuals. Patients with T2DM who lose weight had better metabolic control and fewer complications. Diabetes has been shown to be reversed with large weight decreases of 10-15 kg [3]. Diabetes can impair weight loss for it was shown that people with this disease achieve only half the weight loss of individuals with no diabetes under the same intervention. This occurs because certain antidiabetic drugs exacerbate this problem by inducing weight gain [3]. Despite the advancements in oral and injectable glucose-lowering medications that are currently available, many people with T2DM who have had the disease for a long time eventually need to use insulin [1]. Upon the start of insulin or sulfonylureas (SUs), weight gain is typical; rises of up to 4 kg are frequently noted with insulin and up to 2 kg with SUs [1]. Greater accessibility to obesity-related pharmacotherapeutic drugs would be advantageous for this population [1]. 

A body mass index (BMI) above 30 kg/m^2 ^is a risk factor for T2DM [4]. Decreased insulin production by pancreatic beta cells and peripheral insulin resistance that occur later in the disease course has a vital role in obesity [2]. The incretin system is fundamental to glucose homeostasis, particularly glucagon-like peptide-1 (GLP-1), a gut neuroendocrine hormone stimulating insulin secretion due to oral glucose intake. Individuals with T2DM lack proper incretin response [5]. By restoring the incretin effect and activating GLP-1 receptors in the brain, GLP-1 receptor agonists (GLP-1RAs) lower blood glucose levels and induce satiety, which also reduces appetite, increases fullness, and promotes weight loss [5,6,7]. GLP-1RAs reduce gastric emptying, decreasing food intake and postprandial glucagon and increasing glucose-dependent insulin secretion [2]. 

Traditionally, GLP-1RAs have been prescribed to control blood glucose levels. Recently, however, it has been used to target body weight and satiety [8,9]. Because of the high association of T2DM with obesity, weight loss should be considered to control T2DM [3,10]. GLP-1RAs have emerged as a powerful miracle to treat both T2DM and obesity concurrently [4,7,11].

## Review

Methods

The Preferred Reporting Items for Systematic Reviews and Meta-Analysis (PRISMA) guidelines were used to conduct our systematic literature search. The free full-text articles indexed in PubMed and Google Scholar up until June 28, 2022, were included in this systematic review. The keywords “diabetes,” “type 2 diabetes mellitus,” “glucagon-like peptide-1,” “obesity,” and “weight loss” were the medical subject heading (MeSH) terms used to navigate through the pertinent literature to consider suitable articles. The Boolean operators were incorporated to enhance the precision of relevant articles. Before applying filters, we had 31,328 results from the PubMed search and 22,000 results from the Google Scholar search. After applying the filters, these results decreased to 464 PubMed results and 13,600 Google Scholar results. The detailed search strategy and results are presented in Table 1. 

**Table 1:** Search strategy

Databases	Keywords and regular search strategy	Results before applying filters	Filters	Search results after applying filters
PubMed	Diabetes OR type 2 diabetes mellitus AND glucagon-like peptide-1 ANDobesity AND weight loss	31,328	Free full text, systematic review, clinical trials, 5 years, humans, English language	464
Google Scholar	Diabetes OR type 2 diabetes mellitus AND glucagon-like peptide-1 AND obesity AND weight loss	22,000	1 year	13,600

Study Selection and Eligibility Criteria

The articles were combined in one group in EndNote (Clarivate, London, United Kingdom) and checked for duplicates. Based on their assessments of the study titles and abstracts, two reviewers independently selected the papers that met the requirements for a full-text review. The consensus process was used to settle any disagreements. The selected papers were further examined after applying the eligibility criteria, and then pertinent studies were taken for a full-text review. We included (i) free full texts of literature reviews, systematic reviews, and clinical trials that were relevant to our topic; (ii) papers that were published in English; and (iii) studies that were performed on humans. Studies, such as (i) case reports and commentaries, (ii) studies reporting on animal research, (iii) studies in a language other than English, and (iv) unavailable or retracted articles, were excluded from this review. 

Quality Assessment

We performed a thorough quality assessment for the 13 confirmed articles using two standard tools: the Joanna Briggs Institute (JBI) Checklist (n=9) and Newcastle-Ottawa Checklist (n=4). 

JBI Checklist scores of seven and above out of eight and Newcastle-Ottawa Checklist scores of seven and above out of eight are considered high-quality articles. JBI Checklist scores and Newcastle-Ottawa Checklist scores between four and six out of eight are considered intermediate-quality articles. Moreover, a score below four is deemed (found) to be low quality for both the JBI Checklist and Newcastle-Ottawa Checklist. 

Out of four review articles, two scored eight out of eight and two scored seven out of eight on the JBI Checklist. One executive summary scored eight out of eight on the JBI Checklist. All four observational studies scored seven out of eight on the Newcastle-Ottawa Checklist. All three systematic reviews scored seven out of eight on the Newcastle-Ottawa Checklist. All the articles that satisfied high-quality scores on the JBI Checklist and Newcastle-Ottawa Checklist were included in the review.

Results

**Figure 1:**
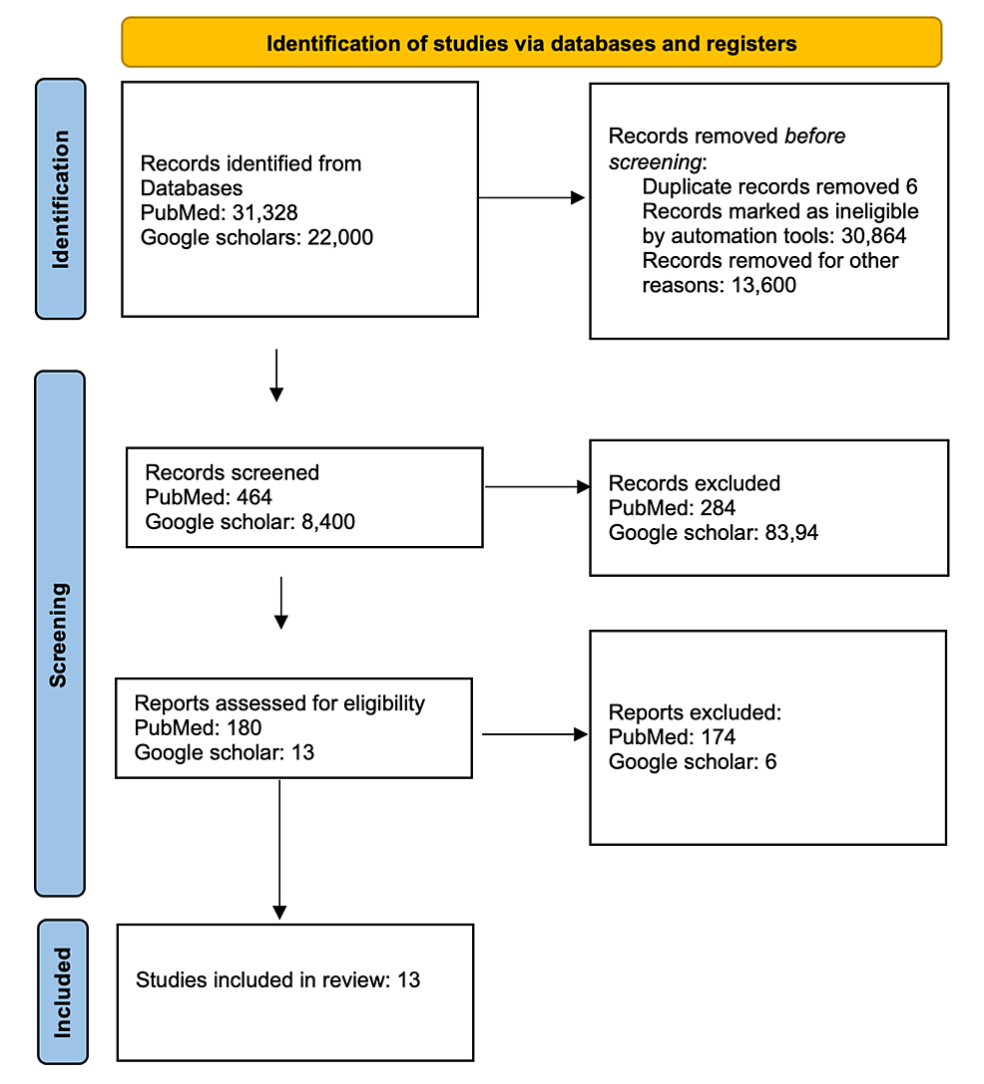
PRISMA diagram detailing the study identification and selection process. PRISMA: Preferred Reporting Items for Systematic Reviews and Meta-analysis

A total of 8,864 relevant articles were found using the advanced search strategy with a combination of regular keywords and MeSH terms, i.e., 464 from PubMed and 8,400 from Google Scholar. Six duplicate articles were removed, and the titles and abstracts of the 8,864 articles were screened. Based on the title and abstract, only 192 articles were relevant to the research topic. Finally, these 192 articles were selected for review. After a full-text review, only 13 were included in our study. The 180 that were excluded either do not meet the inclusion criteria or were inaccessible. We assessed 13 studies for quality appraisal using standardized quality assessment tools, and all articles were qualified after the quality appraisal. The PRISMA flowchart of the literature and search strategy of the studies is shown in Figure 1.

Study Characteristics

Table 2 depicts the data extracted from the studies. Out of the 13 articles, two are systematic reviews and meta-analyses, one is a systematic review, three are randomized clinical trials, one is an executive summary, one is an experimental study, four are reviews, and one is a non-randomized study. The quality assessment tools that were used are the above-mentioned JBI Checklist (case reports/case series) and Newcastle-Ottawa Checklist (observational studies).

**Table 2:** Study characteristics. Y: years; M: male; F: female; N/A: not available; GLP-1RAs: glucagon-like peptide-1 receptor agonists; GLP-1: glucagon-like peptide-1; T2DM: type 2 diabetes mellitus

Sr. no	Author	Year	Study design	Age/Sex	Conclusion
1	Duan K et al. [2]	2022	Systematic review and meta‐analysis	N/A	GLP-1RAs play a vital role in fat redistribution in the body.
2	Li Q et al. [7]	2022	Experimental Study	Adult (>45 Y, M/F)	GLP-1RAs assist in better glycemic control along with weight loss.
3	Klen J et al. [4]	2022	Review article	Adult (>45 Y, M/F)	GLP-1RAs help in weight reduction and treatment of obesity.
4	Zhao S et al. [8]	2022	Review article	Adult (>45 Y, M/F)	These results demonstrate the therapeutic potential of xGLP-1/gastrin for the treatment of obesity and T2DM.
5	Anderson J et al. [5]	2022	Executive summary	Adult (>45 Y, M/F)	Different kinds of GLP-1RAs are available for the treatment of T2DM.
6	Rai AK et al. [11]	2021	Review article	Adult (>45 Y, M/F)	Treatment with GLP-1RAs reduces fat tissues in the body.
7	Valaiyapathi B et al. [12]	2020	Review article	Adolescents (M/F)	The risk factor for T2DM is obesity and related insulin resistance.
8	Garvey WT et al. [1]	2020	Randomized controlled trial	Adult (>45 Y, M/F)	In patients with obesity and T2DM, liraglutide helps in weight reduction and better glycemic control.
9	Tronieri JS et al. [6]	2019	Randomized controlled trial	Adult (>45 Y, M/F)	Intensive behavioral therapy and liraglutide help in better hunger control and fullness.
10	Pratley RE et al. [9]	2019	Randomized study	Adult (>45 Y, M/F)	Efpeglenatid improves glycemic control and weight reduction and later is also controlled in adults with no diabetes.
11	Maula A et al. [3]	2019	Systematic review and meta-analysis	Adult (>45 Y, M/F)	Low-carbohydrate meals along with proper education lead to weight loss in patients with type 2 diabetes.
12	Patel N et al. [10]	2018	Non-randomized study	Adult (18-65 Y, M/F)	EndoBarrier assists in weight loss in patients with diabetes despite using antidiabetic medications.
13	Elder DA et al. [13]	2014	Systematic review	Adolescents (M/F)	Loss of beta cells results in T2DM.

A comprehensive analysis of relevant studies reveals compelling evidence regarding the efficacy of GLP-1RAs in weight management, glycemic control, and the treatment of obesity and diabetes. Duan et al. conducted a meticulous systematic review and meta-analysis, which unequivocally demonstrated the pivotal role of GLP-1RAs in redistributing body fat [2]. 

In a noteworthy experimental study by Li Q et al. involving a diverse adult population (age >45 years, both males and females), GLP-1RAs emerged as a promising adjunct to glycemic control, exhibiting significant potential for weight reduction [7]. 

The comprehensive review article by Klen J et al. focused on adult cohorts (age >45 years, both males and females), further elucidating the positive impact of GLP-1RAs on weight reduction and their efficacy in addressing obesity [4]. 

Zhao S et al. conducted an insightful review article involving adults (age >45 years, both males and females), shedding light on the therapeutic potential of xGLP-1/gastrin for obesity and diabetes treatment. The results underscore the promising prospects for utilizing GLP-1RAs as a therapeutic avenue for combatting both obesity and diabetes [8]. 

In an executive summary by Anderson J et al., which focused on adults (age >45 years, both males and females), the diverse range of available GLP-1RAs for treating T2DM was highlighted. This summary emphasizes the broad array of treatment options available for effectively managing T2DM [5]. 

Rai AK et al. conducted a meticulous review article involving adult populations (age >45 years, both males and females) and established a strong association between GLP-1RA treatment and reduction in adipose tissue. These findings corroborate the therapeutic potential of GLP-1RAs in promoting weight loss [11]. 

Valaiyapathi B et al. conducted a comprehensive review article focusing on adolescents, elucidating the crucial link among obesity, insulin resistance, and the risk of developing T2DM. The study underscores the importance of addressing obesity as a key factor in the prevention and management of T2DM [12]. 

In a significant randomized controlled trial by Garvey WT et al. involving adults (age >45 years, both males and females), liraglutide emerged as a beneficial intervention for weight reduction and improved glycemic control in individuals with obesity and T2DM. These results provide robust evidence supporting the use of GLP-1RA in managing obesity and diabetes [1]. 

Tronieri JS et al. conducted a rigorous randomized controlled trial involving adults (age >45 years, both males and females), showcasing the combined benefits of intensive behavioral therapy and liraglutide in hunger control and satiety. This study presents a compelling case for a comprehensive approach to appetite management and weight loss [6]. 

A significant randomized study by Pratley RE et al. investigated the effects of efpeglenatide on glycemic control and weight reduction, even in adults with no diabetes (age >45 years, both males and females). These findings support the potential broader application of GLP-1RAs in managing glycemic control and weight loss beyond populations with diabetes [9]. 

Maula A et al. conducted a comprehensive systematic review and meta-analysis involving adults (age >45 years, both males and females), underscoring the efficacy of low-carbohydrate meals in conjunction with proper education for weight loss in individuals with T2DM. This study underscores the significance of dietary modifications as an integral component of weight management in individuals with diabetes [3]. 

In a notable non-randomized study by Patel N et al. involving adults (aged 18-65 years, both males and females), the EndoBarrier device showcased its efficacy in facilitating weight loss among patients with diabetes, despite concurrent antidiabetic medication use. These findings highlight the potential of innovative interventions in promoting weight loss in individuals with diabetes [10]. 

Elder DA et al. conducted a systematic review focusing on adolescents, revealing a strong association between the loss of beta cells and the development of T2DM. This study emphasizes the importance of understanding the underlying mechanisms involved in the pathogenesis of T2DM [13].

Discussion

To understand the role of GLP-1RAs in weight loss in T2DM, 13 previously published articles including systematic reviews were included. 

Antidiabetic Drugs and Weight Association

To control blood glucose levels, antidiabetic drugs have been used for a very long time. However, some of these antidiabetic drugs come with some major side effects, such as weight gain. According to a randomized case-control study conducted by Garvey WT et al., starting either insulin or SUs often results in weight gain; an increase of up to 4 kg is typical with insulin and up to 2 kg with SUs. Given that insulin use is associated with weight gain, managing weight may be challenging for those with T2DM and obesity. Furthermore, a systematic review by Duan K et al. showed that people with diabetes have a harder time losing weight; they lose about half as much weight as those without diabetes who get the same intervention, this is further worsened by specific antidiabetic drugs that cause weight gain [1]. 

Zhao S et al emphasized that the T2DM population would benefit from easier access to obesity-related pharmacotherapeutic medications [8]. Because of this, the American Association of Clinical Endocrinologists (AACE) diabetes guidelines, Endocrine Society obesity guidelines, and the most recent European Association for the Study of Diabetes (EASD)/American Diabetes Association (ADA) consensus report suggest that the impact on weight should be considered when deciding on a diabetes treatment. In addition, GLP-1RAs offer an advantage over many other diabetic medications due to their capacity to reduce blood sugar and weight [1,8]. 

In one of the randomized control studies conducted by Tronieri JS et al., liraglutide and semaglutide, which are GLP-1RAs, cause weight reduction by activating GLP-1 receptors in the parts of the brain connected to appetite and food reward. Regarding the doses, it has been allowed to take liraglutide in conjunction with insulin up to 1.8 mg. In addition, it can also be used in a fixed-ratio combination with insulin degludec as a supplement to diet and exercise for the treatment of T2DM [6]. 

Based on the association between antidiabetic drugs and weight gain, it is crucial to consider the impact on weight when deciding on the appropriate treatment for individuals with T2DM and obesity. To address this issue, it is recommended to provide easier access to obesity-related pharmacotherapeutic medications for the T2DM population, as highlighted by Zhao S et al [8]. Among the various antidiabetic medications available, GLP-1RAs offer a distinct advantage due to their dual ability to reduce blood sugar levels and promote weight loss.

Function of GLP-1

According to a study by Klen J et al., the so-called incretin effect shows that oral glucose boosts insulin production more than intravenous glucose infusion does at the same plasma glucose concentration levels. The incretins of the endocrine system, such as glucose-dependent insulinotropic polypeptide (GIP, formerly known as a gastric inhibitory polypeptide) and GLP-1, are involved in the physiological control of glucose homeostasis and postprandial stimulation of insulin production. Incretin secretion is regulated by many interconnected pathways, including many indirect neuro-immuno-hormonal loops and direct interaction with various chemosensors on the brush edge of K and L cells in the gut [4]. 

Furthermore, the release of insulin from pancreatic beta cells is increased by GLP-1. GLP-1 regulates the gene expression of pancreatic cells, stops them from dying, protects them from glucolipotoxicity, and improves their performance. The liver produces less glucose as a result, which lowers blood glucose levels. In addition, it delays stomach emptying and lowers acid output, which reduces appetite and body weight [4]. 

Despite significant inter-individual variation in secretory responses, meta-analyses show no consistent variations in nutrient-induced GLP-1 secretion between healthy and T2DM participants. However, patients with advanced T2DM may have lower amounts of GLP-1 that are active. On the other hand, patients with T2DM experience insulinotropic (and glucagonostatic) effects in response to physiological and pharmacological GLP-1 concentrations. Even in the absence of poor glucose tolerance or T2DM, obesity has been demonstrated to diminish the incretin impact. The incretin effect of GLP-1RAs on pancreatic cells and other peripheral and central mechanisms of action is the foundation of novel therapeutic methods for the treatment of T2DM and obesity [4]. 

Moreover, a randomized study by Pratley RE et al. suggested that the anorexic effects of GLP-1RAs are an independent function playing an important role in weight loss in addition to increasing satiety and decreasing food consumption [9]. 

Further research should be conducted to explore the potential of GLP-1RA therapies in the treatment of T2DM and obesity. Personalized treatment strategies should be developed considering inter-individual variations in GLP-1 secretion. Integrating GLP-1RA therapies into weight management programs could be beneficial. The long-term efficacy and safety of GLP-1RAs should be assessed through large-scale clinical trials. These efforts have the potential to revolutionize T2DM and obesity management, leading to innovative therapeutic interventions and improved patient outcomes.

GLP-1RA Mechanism

Anderson J et al. depict the action of GLP-1, a gut-derived neuroendocrine hormone that promotes insulin secretion in response to oral glucose and plays a key part in the incretin system’s role in glucose homeostasis [5]. The incretin response is lessened in individuals with T2DM. When a GLP-1RA is administered, the incretin action is restored, increasing insulin production and lowering blood sugar levels. GLP-1RAs have a low risk of hypoglycemia because their effects on insulin and glucagon secretion are glucose-dependent. A GLP-1RA should be evaluated before insulin for patients who require injectable therapy because of this property, which is a crucial distinction from insulin [5]. Similarly, Rai AK also highlighted the fact that GLP-1 is released as a result of food consumption in a glucose-dependent manner, which increases insulin secretion. Moreover, it enhances insulin synthesis [11]. 

GLP-1RAs have a distinct glycemic profile in addition to several non-glycemic advantages. One of these is their capacity to encourage satiety, which results in a decrease in food consumption. As a result, these medications encourage weight loss in the majority of T2DM [2,5,9]. 

GLP-1RAs offer a promising therapeutic option for individuals with T2DM. By restoring the impaired incretin response and promoting glucose-dependent insulin secretion, GLP-1RAs effectively lower blood sugar levels without causing hypoglycemia. In addition, these medications promote satiety and weight loss, making them a valuable choice for patients with T2DM struggling with weight management. Overall, GLP-1RAs should be considered an effective and beneficial treatment option for T2DM due to their unique glycemic profile and non-glycemic advantages.

Types of GLP-1RAs and Their Roles

Duan K et al. showed that the number of GLP-1 receptors on intra-abdominal fat cells is significantly higher than that on subcutaneous fat cells in patients with diabetes and obesity, and GLP-1 subsequently causes cell decomposition by activating GLP-1 receptors. This is one of the many hypotheses regarding the mechanism of GLP-1RAs as an influence on fat distribution and weight loss. It was also discovered that low concentrations of GLP-1 (10-12 M) promoted adipocyte synthesis, while high concentrations of GLP-1 (10-10 M) promoted adipocyte decomposition; secondly, GLP-1 acts on the receptor in the nucleus of the solitary tract and reduces appetite and food intake by activating the limbic reward system in the brain; and because both savory and sweet taste buds have GLP-1 receptors, the taste can also reduce appetite; in addition, GLP-1 reduces gastric emptying via binding to GLP-1 receptors in the gastrointestinal (GI) system [2]. 

Furthermore, Anderson J et al. suggested that exenatide and lixisenatide are examples of short-acting GLP-1RAs (dulaglutide, exenatide extended-release, liraglutide, and semaglutide). Although all GLP-1RAs lower blood glucose levels both during fasting and after meals, short-acting medicines have a stronger effect on postprandial glucose, while long-acting drugs have a greater impact on fasting plasma glucose. Considerations for treatment individualization include additional variations among the GLP-1RAs, such as dose frequency, method of administration, GI adverse effects, and cardiovascular (CV) advantages [5]. 

The impact of GLP-1RAs on blood glucose control varies depending on how long they remain active. Gastric emptying is more substantially delayed by short-acting GLP-1RAs, which have a greater impact on postprandial blood sugar [4]. 

Efpeglenatide, administered once a week or once every two weeks, significantly lowered body weight compared to placebo and enhanced glycemic and lipid parameters. Adults who used it to control their weight and did not have diabetes indicated that it was well tolerated [9]. 

The diverse roles and mechanisms of GLP-1RAs highlight their potential for personalized treatments in diabetes management. Specific considerations include the choice between short-acting (e.g., exenatide and lixisenatide) and long-acting (e.g., dulaglutide, liraglutide, and semaglutide) GLP-1RAs, as their effects on blood glucose control differ. Short-acting agents demonstrate a stronger impact on postprandial glucose levels, while long-acting ones have a greater effect on fasting plasma glucose. In addition, the choice of GLP-1RAs should consider factors, such as dose frequency, method of administration, GI adverse effects, and CV benefits. Furthermore, emerging agents like efpeglenatide show promise in weight control and glycemic improvement, even in individuals with no diabetes. These findings emphasize the importance of tailoring GLP-1RA therapies based on individual needs and characteristics.

GLP-1RA Side Effects

Because GLP-1 is a neuroendocrine hormone generated from the gut, majority of patients treated with a GLP-1RA have one or more GI adverse effects early on in the course of treatment - typically nausea and/or vomiting. It is crucial to inform patients that early satiety, which may be mistaken for nausea when used in GLP-1RA therapy, is promoted. Therefore, it is important to advise people to stop eating as soon as they start to feel full. To minimize dehydration, especially in those with or at risk for kidney issues, anyone who develops prolonged GI side effects while receiving GLP-1RA medication may be urged to contact their doctor [5]. 

The studies demonstrated that exenatide (in its original weekly formulation) was one of the first GLP-1RA to cause injection site responses. Although uncommon allergic reactions can happen, these reactions have fortunately been proven to be unusual with the other GLP-1RAs. 

Preclinical studies using non-human primate rats receiving a GLP-1RA revealed medullary thyroid C-cell malignancies [4,5]. 

Data proving that GLP-1RA treatment causes pancreatitis are yet lacking. In actuality, ongoing studies, particularly the several multiyear cardiovascular outcome trials (CVOTs) carried out recently, have not shown any increased risk of pancreatitis with the use of GLP-1RAs. It is unclear whether using GLP-1RAs increases the chance of developing pancreatitis in people with a history of the condition. The GLP-1RAs should be stopped and proper care should be started in the event that pancreatitis is suspected in a patient receiving treatment with a substance from this family of medications [4,5]. 

When prescribing GLP-1RAs, it is important to inform patients about the potential GI adverse effects, such as early satiety, nausea, and vomiting. Patients should be advised to stop eating when feeling full to minimize the discomfort associated with GLP-1RA therapy. It is crucial for individuals experiencing prolonged GI side effects to seek medical attention, especially those with kidney issues to prevent dehydration. Although injection site reactions and allergic responses have been observed with some GLP-1RAs, these occurrences are infrequent. While preclinical studies indicate a potential risk of medullary thyroid C-cell malignancies, the evidence regarding GLP-1RA-induced pancreatitis remains inconclusive. Ongoing studies, including CVOTs, have not demonstrated an increased risk of pancreatitis associated with GLP-1RA use. However, if pancreatitis is suspected in a patient receiving GLP-1RA treatment, discontinuation of the medication and appropriate care should be initiated. 

Limitations and strengths

We have conducted a systematic review that has certain limitations. First, the studies were conducted in three databases in only the English language. The studies might have discrepancies regarding information in other languages. Second, we used data available only in free full text, so important information during the screening process might be left out. Lastly, we have not included studies on animals, so recently updated information might be excluded that could have affected our research. 

However, our review also shows many strengths. There are significant data available on the human population that emphasize the role of GLP-1RAs in promoting weight loss in patients with T2DM and obesity. This systematic review further confirms the significance of GLP-1RAs and advises selective prescription of these drugs to satisfy patient needs.

## Conclusions

This systematic review confirms that GLP-1RAs demonstrate significant potential in promoting weight loss in individuals with T2DM and obesity. These medications offer an advantage over other antidiabetic drugs due to their ability to reduce blood sugar levels and facilitate weight loss. Despite the potential GI side effects, GLP-1RAs are generally well-tolerated. Based on the evidence presented, it is recommended that healthcare professionals consider weight reduction when choosing antidiabetic medications for individuals with T2DM and obesity. GLP-1RAs, such as short-acting and long-acting options, have demonstrated efficacy in reducing blood sugar levels and promoting weight loss. Individualized treatments should take into account variations among GLP-1RAs, including dose frequency, method of administration, GI adverse effects, and cardiovascular benefits. Early GI side effects may occur, necessitating close monitoring and management. Further research should focus on long-term outcomes and potential interactions with comorbidities. By following these guidelines, healthcare professionals can optimize the use of GLP-1RAs as a valuable therapeutic option for weight loss in T2DM and obesity management.
